# Bile acid distributions, sex-specificity, and prognosis in colorectal cancer

**DOI:** 10.1186/s13293-022-00473-9

**Published:** 2022-10-23

**Authors:** Yuping Cai, Xinyi Shen, Lingeng Lu, Hong Yan, Huang Huang, Patricia Gaule, Engjel Muca, Casey M. Theriot, Zahra Rattray, Nicholas J. W. Rattray, Jun Lu, Nita Ahuja, Yawei Zhang, Philip B. Paty, Sajid A. Khan, Caroline H. Johnson

**Affiliations:** 1 Department of Environmental Health Sciences, Yale School of Public Health, Yale University, New Haven, CT 06510 USA; 2 Interdisciplinary Research Center on Biology and Chemistry, Shanghai Institute of Organic Chemistry, Chinese Academy of Sciences, Shanghai, 200032 China; 3 Department of Chronic Disease Epidemiology, Yale School of Public Health, Yale University, New Haven, CT 06510 USA; 4 Department of Pathology, Yale University School of Medicine, New Haven, CT 06510 USA; 5 Department of Surgery, Memorial Sloan Kettering Cancer Center, New York, NY USA; 6 North Caroline State University, Raleigh, NC USA; 7 Strathclyde Institute of Pharmacy and Biomedical Sciences, University of Strathclyde, Glasgow, G4 0RE UK; 8 Yale Stem Cell Center, Yale University School of Medicine, New Haven, CT 06520 USA; 9 Department of Genetics, Yale University School of Medicine, New Haven, CT 06520 USA; 10 Department of Pathology, Yale University School of Medicine, New Haven, CT 06510 USA; 11 Department of Surgery, Division of Surgical Oncology, Yale University School of Medicine, New Haven, CT 06510 USA; 12 National Cancer Center, National Clinical Research Center for Cancer, Cancer Hospital, Chinese Academy of Medical Sciences and Peking Union Medical College, Beijing, China

## Abstract

**Background:**

Bile acids are known to be genotoxic and contribute to colorectal cancer (CRC). However, the link between CRC tumor bile acids to tumor location, patient sex, microbiome, immune-regulatory cells, and prognosis is not clear.

**Methods:**

We conducted bile acid analysis using targeted liquid chromatography–mass spectrometry (LC–MS) on tumor tissues from CRC patients (*n* = 228) with survival analysis. We performed quantitative immunofluorescence (QIF) on tumors to examine immune cells.

**Results:**

Twelve of the bile acids were significantly higher in right-sided colon tumors compared to left-sided colon tumors. Furthermore, in male patients, right-sided colon tumors had elevated secondary bile acids (deoxycholic acid, lithocholic acid, ursodeoxycholic acid) compared to left-sided colon tumors, but this difference between tumors by location was not observed in females. A high ratio of glycoursodeoxycholic to ursodeoxycholic was associated with 5-year overall survival (HR = 3.76, 95% CI = 1.17 to 12.1, *P* = 0.026), and a high ratio of glycochenodeoxycholic acid to chenodeoxycholic acid was associated with 5-year recurrence-free survival (HR = 3.61, 95% CI = 1.10 to 11.84, *P* = 0.034). We also show correlation between these bile acids and FoxP3 + T regulatory cells.

**Conclusions:**

This study revealed that the distribution of bile acid abundances in colon cancer patients is tumor location-, age- and sex-specific, and are linked to patient prognosis. This study provides new implications for targeting bile acid metabolism, microbiome, and immune responses for colon cancer patients by taking into account primary tumor location and sex.

**Supplementary Information:**

The online version contains supplementary material available at 10.1186/s13293-022-00473-9.

## Background

Colorectal cancer (CRC) is the third most diagnosed cancer in both males and females in the United States, and it is also the third leading cause of cancer-related deaths [1, 2]. Recent decades have seen a shift in the incidence of tumors by anatomic location, with an increasing proportion arising on the right side of the colon [2, 3]. Right-sided colon cancer (RCC) is categorized as a primary tumor that presents in the cecum, ascending and hepatic flexure colon, whereas left-sided colon cancer (LCCs) presents in the splenic flexure, descending, sigmoid, and rectosigmoid colon. The CRC incidence rate is lower in females (34.0 per 100,000 population) than in males (44.4 per 100,000 population). However, if we examine this rate by lesion location in the colon, various studies have shown that patients with RCCs are more likely to be females [4, 5]. An analysis of stage I–III patients from Surveillance, Epidemiology, and End Results (SEER) data showed that 62% of RCCs were from females, whereas for LCCs there was a more equal distribution between the sexes (52% from females) [4]. RCC patients are older, and females live longer than males, thus this sex-difference in RCC may be reflection of ageing-related differences also. However, the sex-specific difference in the anatomic location of CRC lesions is concerning for females, as patients with RCC have a higher mortality compared with those with LCC [6].

The reason for differences in clinical outcome based on anatomic location of the tumor is not known. In addition, it is not known why females have a higher incidence of RCCs. Several biological differences exist between RCCs and LCCs that could contribute, including frequency of genetic mutations, methylation, and immunogenicity [7–9]. Also RCCs have a higher preponderance of tumors that develop through the serrated neoplastic pathway resulting in flatter polyps that are more difficult to identify at early clinical stages during colonoscopy screening, however patients with RCC still have a higher mortality than LCCs after adjusting for stage of diagnosis [6]. Beyond genetic variation, the microbiome which is known to differ by diversity and structure between these anatomical regions could contribute to the heterogeneity [10, 11]. RCCs have also been shown to display invasive polymicrobial bacterial biofilms, which are intensive aggregates of bacteria that can invade the mucus layer of colon and interact with the epithelial cells causing a pro-carcinogenic environment [12, 13]. In addition to these findings, we previously revealed a difference in metabolome and transcriptome between RCC and LCC tumors from CRC patients, showing microbial metabolites in RCCs, and a nutrient deplete phenotype in female patients with RCC [14–16].

Bile acids are products of cholesterol metabolism metabolized by intestinal microbiota into genotoxic compounds, and recently they have been linked to immune responses [17]. Primary bile acids such as chenodeoxycholic acid (CDCA) and taurocholic acid (TCA) have been shown to induce accumulation of natural killer T cells against both primary and metastatic liver tumors, and microbial conversion of these bile acids to secondary bile acids reverses this effect [18]. An anti-inflammatory role has been observed for metabolites of lithocholic acid (LCA; a secondary bile acid) wherein both 3-oxoLCA and isoalloLCA modulate T_H_17 cell and T_reg_ cell differentiation [19]. For healthy adults, the right side of the colon has been shown to have higher abundances of primary bile acids cholic acid (CA) and CDCA [20, 21], and sex-related differences have been reported in bile acid abundances [22]. However, for CRC, the differences in bile acid abundance between anatomic locations of the colon remains poorly characterized. Given that bile acids have been shown to regulate immune responses in other gastrointestinal cancers, it will be important to understand their roles in patient outcomes in CRC, particularly with regard to their association with anatomic location, sex, and patient prognosis.

In this study, we hypothesize that there are critical biological differences in bile acid abundances between primary tumors based on tumor anatomic location and patient sex, which correlate with patient prognosis. Furthermore, we examined whether regulatory T cells are linked to bile acid abundances. This study has revealed a critical insight into anatomic location- and sex-specific differences of bile acids and their associated biological responses. As bile acids are known to be genotoxic and are reported to have immunomodulatory roles in cancer, our study shows that additional patient and tumor-related factors that should be considered when targeting bile acids in colon cancer.

## Results

### Differences in bile acid levels and microbiome based on primary tumor location for colon cancer

High-resolution quantification was carried out for measurement of 14 bile acids including primary bile acids, secondary bile acids, and their conjugated forms (taurine and glycine conjugated) (Additional file 1: Table S1) using a time-of-flight multiple reaction monitoring mass spectrometry (ToF-MRM-MS) approach. The analysis was conducted on resected primary tumor tissues from a prospectively collected cohort of patients with stage I–III RCC or LCC (*n* = 228). Tumors from two age groups (≥ 55 years old (*n* = 197) and < 55 years old (*n* = 31)) of patients with colon cancer were analyzed to examine additional age-specific effects. The detailed information of the clinical cohort is listed in Additional file 1: Table S2.

**Fig. 1:**
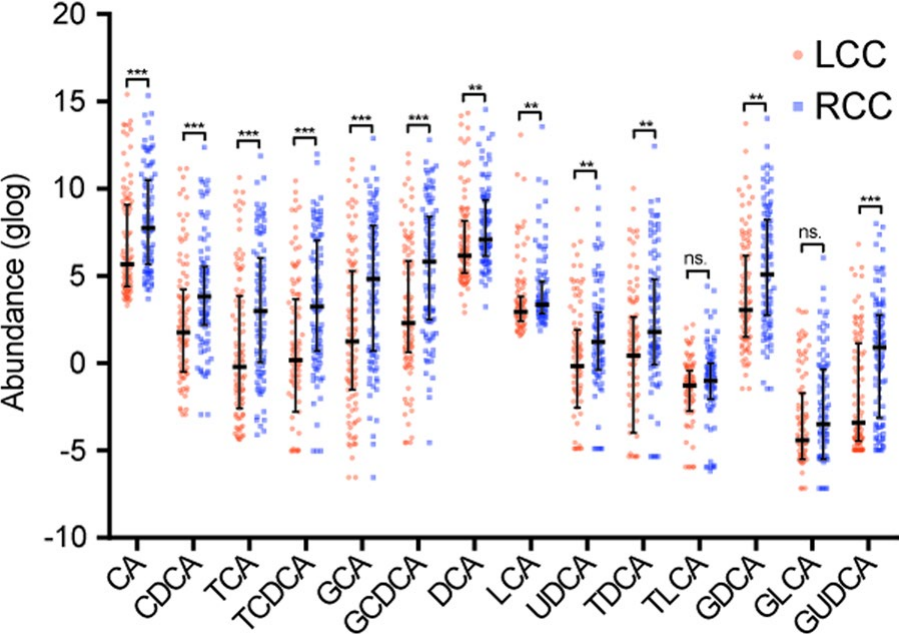
Differences in tumor tissue bile acids between left-sided colon cancers (LCCs) and right-sided colon cancers (RCCs) from patients ≥ 55 years old. Stages I–III combined (LCC *n* = 99, RCC *n* = 98). Data represent median with interquartile range. Nonparametric Wilcoxon Mann–Whitney *U* test, *p* values adjusted for false discovery rates (FDR) (Benjamini–Hochberg). *p < 0.05, **p < 0.01, ***p < 0.001, *ns.*  not significant; *CA* cholic acid, *CDCA* chenodeoxycholic acid, *TCA* taurocholic acid, *TCDCA* taurochenodeoxycholic acid, *GCA* glycocholic acid, *GCDCA* glycochenodeoxycholic acid, *DCA* deoxycholic acid, *LCA* lithocholic acid, *UDCA* ursodeoxycholic acid, *TDCA* taurodeoxycholic acid, *TLCA* taurolithocholic acid, *GDCA* glycodeoxycholic acid, *GLCA* glycolithocholic acid, *GUDCA* glycoursodeoxycholic acid, *DCA* deoxycholic acid, *TDCA* taurodeoxycholic acid, *GDCA* glycodeoxycholic acid

Bile acid abundances were initially compared between RCC and LCC tissues from patients ≥ 55 years old (stages I–III) to identify primary tumor anatomic location-specific bile acids. Twelve out of 14 bile acids measured were significantly upregulated in RCCs compared to LCCs (*n* = 197) (Fig. 1). Primary bile acids CA, CDCA, and conjugated primary bile acids TCA, glycocholic acid (GCA), taurochenodeoxycholic acid (TCDCA) and glycochenodeoxycholic acid (GCDCA) were increased in RCCs compared to LCCs (*P* < 0.05). Similar results were observed for secondary bile acids; deoxycholic acid (DCA), lithocholic acid (LCA), ursodeoxycholic acid (UDCA), and conjugated secondary bile acids taurodeoxycholic acid (TDCA), glycodeoxycholic acid (GDCA), and glycoursodeoxycholic acid (GUDCA) (*P* < 0.05). The taurine and glycine conjugates of LCA, taurolithocholic acid (TLCA) and glycolithocholic acid (GLCA), had no significant difference between patients with RCC and LCC. When examining the differences in bile acid abundances between RCCs and LCCs in patients < 55 years old (*n* = 31), the concentrations of bile acids were independent of tumor location (Additional file 1: Fig. S1).

We further explored the influence of tumor stage in patients > 55 years old. Tumor location differences were seen within stage I (*n* = 47) and stage II (*n* = 86) tumors (Additional file 1: Fig. S2A and 2B), but not in stage III tumors (*n* = 64) (Additional file 1: Fig. S2C). For stage I tumors, no anatomic location differences were observed for unconjugated primary bile acids (CA and CDCA) and secondary bile acids (DCA and LCA) (Additional file 1: Fig. S2A). However, all amino acid-conjugated forms of primary bile acids TCA, TCDCA, GCA, GCDCA, and GUDCA were increased in RCCs compared to LCCs. In addition, UDCA was significantly higher in RCCs compared to LCCs. Taurine-conjugated secondary bile acids TDCA and TLCA were upregulated in RCCs, but glycine-conjugated forms were not different between LCCs and RCCs. Of note, we identified that the dependence of tumor location for bile acid abundances in stage II tumors was the same as in stage I–III tumors combined (Additional file 1: Fig. S2B). Collectively, higher abundances of bile acids were seen in RCCs compared to LCCs for patients ≥ 55 years old, however the composition of these bile acids differed by tumor stage.

We also explored whether there were differences in tumor microbiome by tumor anatomic location using Kraken TCGA microbial detection, as previously described [23]. Using publicly available data obtained from TCGA COAD, we compared the abundance of bacteria in tumors from RCCs (*n* = 136) to LCCs (*n* = 262), RCC tissues had a significantly different bacterial pattern with higher abundances of *Firmicutes* and *Proteobacteria* (bacterial classes enriched in bile salt hydrolase (BSH) that metabolize bile acids [24, 25] (Additional file 2: Table S3)). We show that the top 5 genera with FDR 0.047 included *Faecalibacterium* (log(Fold change) = 1.40), *Coprococcus* (log(Fold change) = 0.92), *Dorea* (log(Fold change) = 1.02), *Luteibacter* (log(Fold change) = 0.35) which were all increased in RCCs compared to LCCs, and *Sorangium* (log(Fold change) = − 0.34) which was decreased (Additional file 1: Fig. S3). The approach we used for microbial sequencing had some limitations. TCGA datasets are based on polyA-enriched specimens, which could potentially limit the number of microbiota that can be examined.

### Sex-related differences in bile acids stratified by anatomic location

To investigate whether bile acid distributions of primary colon tumors differs by tumor location and sex of the patients, we initially compared bile acid abundances directly between males and females for all tumors (Additional file 1: Fig. S4A), and then for RCCs and LCCs separately (Additional file 1: Figs. S4B-C). For all comparisons, there were no significant differences in bile acid levels between males and females. We then examined differences in bile acids between RCCs and LCCs for females and males separately to determine whether sex-differences exist in the distribution of bile acids in the colon. For patients aged ≥ 55 years old, sex-specific differences in secondary bile acids were identified when all three stages (I, II, and III) were integrated, wherein DCA, LCA, and UDCA were increased only in male patients with RCC compared to males with LCC, but not in females with RCC compared to LCCs (Additional file 1: Table S4). Primary bile acids (CA and CDCA), taurine-conjugated primary bile acids (TCA and TCDCA), and glycine-conjugated primary bile acids (GCA and GCDCA) were increased in RCC compared to LCCs for both females and males (Additional file 1: Table S4). For patients aged < 55 years old, no significant differences were seen between RCC and LCC in either females or in males. However, the results indicated a trend of higher bile acid abundance in males with RCC compared with LCCs from male patients. This trend was not seen in females.

Stratification by tumor stage revealed that additional sex-specific differences in bile acid abundances were present in stage II tumors (Table 1). Secondary bile acids DCA, LCA, and UDCA were increased only in male patients with RCC compared to LCC, similar to the result observed by combining data from all three stages. However, TDCA and GDCA were only increased in females with RCC compared with LCCs, and no differences were seen between male patient tumors by tumor location for these metabolites. In addition, specific primary bile acids such as CDCA and GCA, and glycine-conjugated secondary bile acid GUDCA were upregulated only in males with RCC compared with LCC. Combined, these results show sex- and anatomic location-specific differences in bile acid abundances in colon tumor tissues.

**Table 1:** Sex differences in bile acid levels by comparing stage II RCC and LCC from patients aged ≥ 55 years

		Females (RCC vs LCC)		Males (RCC vs LCC)	
		FC^a^	*P* value^b^	FC^a^	*P* value^b^
Primary BAs	CA	7.4	0.036	7.4	0.011
	CDCA*	–	ns	6.5	0.011
Taurine-conjugated primary BAs	TCA	33.1	0.036	14.0	0.041
	TCDCA	16.7	0.036	14.2	0.012
Glycine-conjugated primary BAs	GCA*	–	ns	7.8	0.020
	GCDCA	14.6	0.041	10.6	0.011
Secondary BAs	DCA*	–	ns	3.4	0.011
	LCA*	–	ns	1.6	0.041
	UDCA*	–	ns	3.4	0.020
Taurine-conjugated secondary BAs	TDCA*	7.4	0.041	–	ns
	TLCA	–	ns	–	ns
Glycine-conjugated secondary BAs	GDCA*	19.0	0.036	–	ns
	GLCA	–	ns	–	ns
	GUDCA*	–	ns	23.3	0.011

^a^FC = Fold change, fold change calculated by dividing median value of right-sided colon cancer (RCC) by median value of left-sided colon cancer (LCC). ^b^*P*-values estimated by the Mann–Whitney *U* test adjusted for false discovery rates (FDR) (Benjamini–Hochberg). ns = not significant. ^*^Bile acids identified with sex-specific differences. CA, cholic acid; CDCA, chenodeoxycholic acid; TCA, taurocholic acid; TCDCA, taurochenodeoxycholic acid; GCA, glycocholic acid; GCDCA, glycochenodeoxycholic acid; DCA, deoxycholic acid; LCA, lithocholic acid; UDCA, ursodeoxycholic acid; TDCA, taurodeoxycholic acid; TLCA, taurolithocholic acid; GDCA, glycodeoxycholic acid; GLCA, glycolithocholic acid; GUDCA, glycoursodeoxycholic acid

### Bile acid abundances in tumors have associations to prognosis in colon cancer

We next examined the associations between bile acid levels and patient prognosis. In Fig. 1, we show that 12 bile acids are significantly increased in RCCs from patients ≥ 55 years old, and notably, patients with RCCs are known to have a poorer prognosis. However, this difference in bile acid abundances between RCCs and LCCs was not conserved during subgroup analysis by stage or sex. To investigate the impact of bile acid abundances in tumor tissue on patient outcomes, we carried out an association analyses using multivariable Cox proportional hazard (PH) regression models. Given that conjugated bile acids can undergo deconjugation by intestinal bacteria, we also included the ratio of conjugated bile acids to unconjugated bile acids for association analyses. The models were established by including bile acids and ratios and excluding those that were positively correlated (Additional file 1: Fig. S5). Due to a limited number of death events compared to number of patients in each subgroup of patients by anatomic location, sex and stage, Cox PH models failed to give reliable estimates in subgroup analysis. We added these as covariates in our model along with age and chemotherapy use. In patients aged ≥ 55 years old, a high ratio of GUDCA to UDCA was significantly associated with shortened 5-year overall survival (OS) (HR = 3.76, 95% CI = 1.17 to 12.1, *P* = 0.026) adjusted for clinical covariates and other bile acids/ratios, however no other bile acids were associated with OS (Table 2). A high ratio of GCDCA to CDCA was observed with shorter 5-year recurrence-free survival (RFS) (HR = 3.61, 95% CI = 1.10 to 11.84, *P* = 0.034) adjusted for clinical covariates and other bile acids/ratios (Table 3). As mentioned, further stratification by tumor location was not possible as there were low sample numbers in the patients that had recurrence (LCC; *n* = 19, RCC; *n* = 11) compared to much larger samples numbers in those that did not have recurrence (LCC; *n* = 80, RCC; *n* = 87). A relatively large number of variables were considered in the model, therefore it was not possible to accurately determine whether these bile acid ratios associated with patient prognosis by sidedness.

**Table 2:** Multivariable Cox regression associating 17 bile acid levels or ratio of conjugated bile acids to unconjugated bile acids and 5-year overall survival for patients ≥ 55 years old (*n* = 197) adjusting for age, sex, tumor anatomic location, stage, and chemotherapy use

Bile acid/ratio	HR	CI95	*P* value
CA	0.94	0.37–2.42	0.905
GCA	1.18	0.34–4.06	0.796
DCA	0.84	0.29–2.41	0.742
LCA	1.11	0.48–2.59	0.807
UDCA	1.08	0.41–2.89	0.873
TDCA	1.29	0.33–5.03	0.718
TLCA	1.73	0.63–4.75	0.290
GDCA	0.98	0.23–4.11	0.975
TCA/CA	2.85	0.79–10.29	0.109
TCDCA/CDCA	0.45	0.17–1.22	0.116
GCA/CA	0.36	0.09–1.56	0.173
GCDCA/CDCA	2.2	0.83–5.86	0.113
TDCA/DCA	0.81	0.21–3.05	0.752
TLCA/LCA	0.4	0.12–1.38	0.149
GDCA/DCA	0.92	0.28–2.99	0.885
GLCA/LCA	0.76	0.32–1.82	0.537
GUDCA/UDCA	3.76	1.17–12.1	0.026

*HR*  hazard ratio, *CI*  confidence interval, *CA* cholic acid, *CDCA* chenodeoxycholic acid, *TCA* taurocholic acid, *TCDCA* taurochenodeoxycholic acid, *GCA* glycocholic acid, *GCDCA* glycochenodeoxycholic acid, *DCA* deoxycholic acid, *LCA* lithocholic acid, *UDCA* ursodeoxycholic acid, *TDCA* taurodeoxycholic acid, *TLCA* taurolithocholic acid, *GDCA* glycodeoxycholic acid, *GLCA* glycolithocholic acid, *GUDCA* glycoursodeoxycholic acid

**Table 3:** Multivariable Cox regression associating 17 bile acid abundances or ratios to 5-year recurrence-free survival (RFS) for patients ≥ 55 years old (*n* = 197) adjusting for age, sex, tumor anatomic location, stage, and chemotherapy use

Bile acid/ratio	HR	CI95	*P* value
CA	0.65	0.23–1.80	0.405
GCA	2.16	0.55–8.49	0.269
DCA	0.52	0.16–1.66	0.270
LCA	2.47	0.89–6.83	0.083
UDCA	0.75	0.25–2.27	0.616
TDCA	1.26	0.30–5.25	0.750
TLCA	2.23	0.78–6.41	0.136
GDCA	0.42	0.08–2.36	0.326
TCA/CA	0.79	0.22–2.86	0.724
TCDCA/CDCA	0.4	0.12–1.30	0.127
GCA/CA	3.22	0.71–14.53	0.128
GCDCA/CDCA	3.61	1.10–11.84	0.034
TDCA/DCA	0.92	0.25–3.39	0.896
TLCA/LCA	1.82	0.54–6.13	0.332
GDCA/DCA	0.43	0.10–1.84	0.257
GLCA/LCA	0.55	0.22–1.39	0.207
GUDCA/UDCA	0.62	0.20–1.86	0.392

*HR*  hazard ratio, *CI* confidence interval, *CA* cholic acid, *CDCA* chenodeoxycholic acid, *TCA* taurocholic acid, *TCDCA* taurochenodeoxycholic acid, *GCA* glycocholic acid, *GCDCA* glycochenodeoxycholic acid, *DCA* deoxycholic acid, *LCA* lithocholic acid, *UDCA* ursodeoxycholic acid, *TDCA* taurodeoxycholic acid, *TLCA* taurolithocholic acid, *GDCA* glycodeoxycholic acid, *GLCA* glycolithocholic acid, *GUDCA* glycoursodeoxycholic acid

We next examined whether any other tumor-related metabolites were correlated with bile acids levels, to potentially uncover any novel relationships between tumor metabolism and bile acid regulation. We carried out Pearson correlation analysis between each bile acid and 93 metabolites that we previously identified in these tumors in an untargeted metabolomics study [14]. We observed that only glucuronic acid was positively correlated with taurine and glycine-conjugated bile acids, and only in RCCs (Table 4). In LCCs, glucuronic acid is positively correlated with CA only (Additional file 1: Table S5).

**Table 4:** Pearson correlation of glucuronic acid to bile acid abundances in patients with right-sided colon cancer ≥ 55 years old (*n* = 98)

Bile acid	*R*	*P* value
CA	0.251	ns
CDCA	0.083	ns
GCA	0.735	< 0.001
DCA	0.063	ns
LCA	0.060	ns
UDCA	0.022	ns
TDCA	0.680	< 0.001
TLCA	0.555	< 0.001
GDCA	0.671	< 0.001
GLCA	0.134	ns
TCA	0.680	< 0.001
TCDCA	0.584	< 0.001
GCA	0.735	< 0.001
GCDCA	0.673	< 0.001

*R* = Pearson correlation coefficient, *p* = adjusted for false discovery rates (FDR) (Benjamini–Hochberg). *CA* cholic acid, *CDCA* chenodeoxycholic acid, *TCA* taurocholic acid, *TCDCA* taurochenodeoxycholic acid, *GCA* glycocholic acid, *GCDCA* glycochenodeoxycholic acid, *DCA* deoxycholic acid, *LCA* lithocholic acid, *UDCA* ursodeoxycholic acid, *TDCA* taurodeoxycholic acid, *TLCA* taurolithocholic acid, *GDCA* glycodeoxycholic acid, *GLCA* glycolithocholic acid, *GUDCA* glycoursodeoxycholic acid

### Glycine-conjugated bile acids associate with T cells

**Fig. 2:**
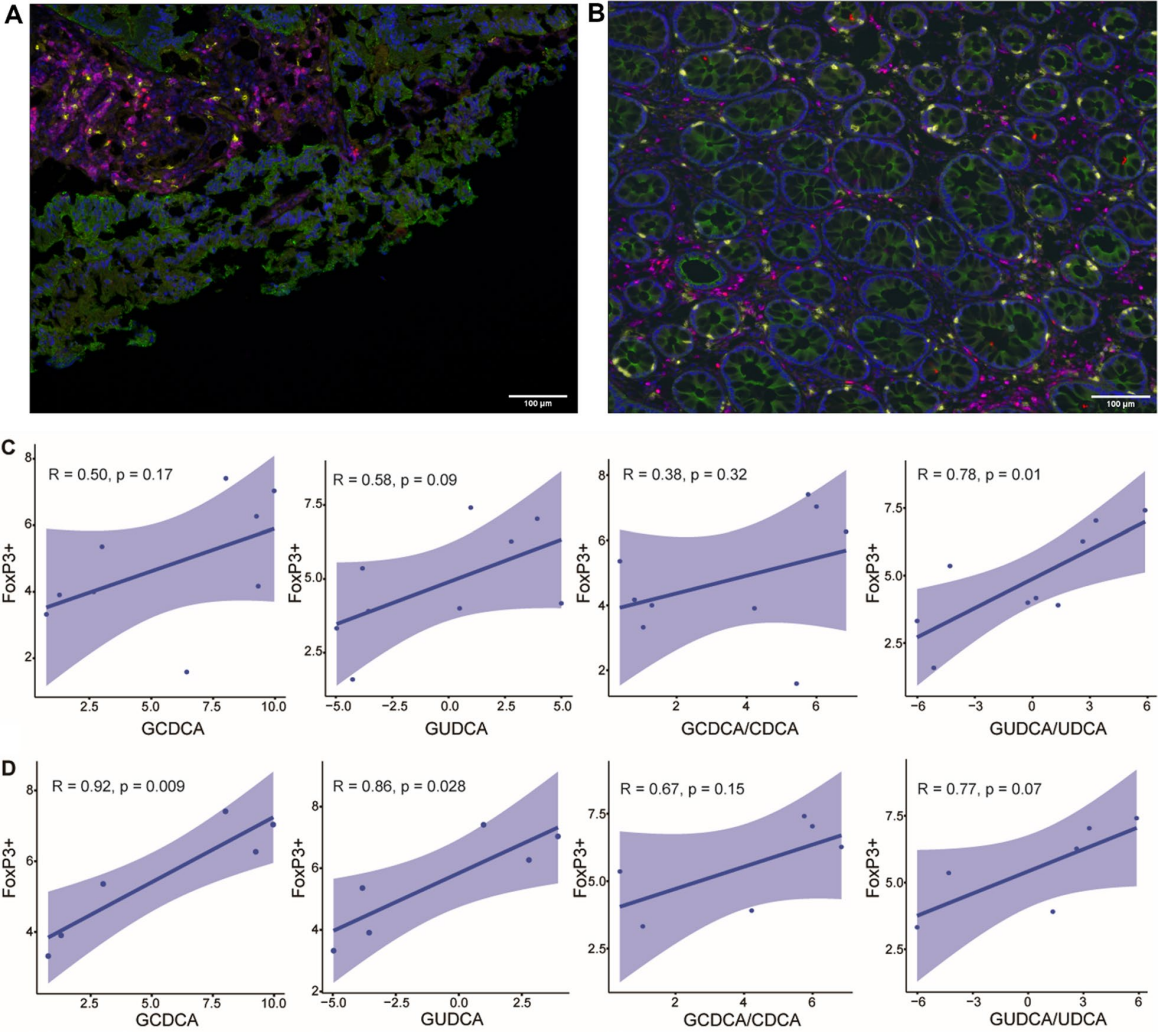
Linear regression of T cell abundances (CD8 + and CD4 + FoxP3 +) quantified by quantitative immunofluorescence (QIF) against relative abundance of bile acids linked to poorer clinical outcomes. **A** Representative right-sided colon tumor tissue stained and imaged, blue = DAPI; nuclei, green = cytokeratin, yellow = CD8 + T cells, magenta = CD4 + T cells, Red = FoxP3 + T cells, scale bar 100 μM. **B** Representative histopathologic assigned normal colon tissue from a right-sided colon cancer patient, blue = DAPI; nuclei, green = cytokeratin, yellow = CD8 + T cells, magenta = CD4 + T cells, Red = FoxP3 + T cells, scale bar 100 μM. **C** Correlation between CD4 + FoxP3 + and glycine-conjugated bile acids stage II tumors (*n* = 10). **D** Correlation between CD4 + FoxP3 + and glycine-conjugated bile acids stage II right-sided colon tumors (*n* = 6). * *P* < 0.05, 95% confidence bands shown

To examine whether the bile acid levels could be linked to immune responses associated with prognosis, we conducted QIF (quantitative immunofluorescence) on tumors from patients; Fig. 2A and B shows representative staining of immune cells in a right-sided colon tumor and right-sided normal colon tissue taken from a RCC patient. Table 5 shows the correlation between GUDCA, GCDCA, the ratios of GCDCA to CDCA, and GUDCA to UDCA against CD8 + and FoxP3 + Tregs for ten tumor tissues analyzed. The ratio of GUDCA to UDCA was positively associated with FoxP3 + Tregs (*R* = 0.78, *p* = 0.013), however the other bile acid levels were not correlated with T cells (Fig. 2C). We also analyzed the correlation between CD4 + T cells and GUDCA/UDCA showing a positive trend for all ten stage II tumors; however, it was not statistically significant (Additional file 1: Fig. S6). As the distribution of bile acids are higher in RCCs compared to LCCs, and have sex-related differences in distribution, we also examined their correlations to T cells in RCCs only (*n* = 6). Figure 2D and Additional file 1: Table S6 show that the levels of GUDCA and GCDCA in RCCs were positively correlated with FoxP3 + Tregs (*R* = 0.86, *p* = 0.028, *R* = 0.92, *p* = 0.009, respectively), whereas the ratio of the other bile acids were not correlated. Furthermore, CD4 + T cells again displayed a positive trend with GUDCA/UDCA in stage II RCCs (Additional file 1: Fig. S6). These results show that the glycine-conjugated bile acids that are linked to poorer OS and RFS are positively associated with FoxP3 + Treg cells in colon cancer, particularly higher levels of GUDCA and GCDCA in RCCs. Of note, only stage II tumor tissues were examined as tumor location differences were observed in bile acid levels within stage II tumors (Additional file 1: Fig. S2B).

**Table 5:** Correlations between prognosis-linked bile acid levels and T cell abundances in tissues (*n* = 10). Tumor tissues from stage II patients, 6 RCCs and 4 LCCs

Bile acid	CD8 +		CD4 + FoxP3 +	
	*R*	*P* value	*R*	*P* value
GUDCA	− 0.16	0.66	0.58	0.10
GCDCA	− 0.16	0.65	0.50	0.17
GCDCA/CDCA	− 0.23	0.53	0.38	0.32
GUDCA/UDCA	0.17	0.63	0.78	0.01

## Discussion

Our study focused on the role of bile acids in CRC. Bile acids are genotoxins, tumor promotors, and are at found at higher abundances in the right colon. Bile acids are not only end products of cholesterol metabolism that facilitate nutrient absorption, but are also signaling molecules modulating physiological status. As higher abundances of estradiol have been shown to lower serum cholesterol and bile acid abundances [26], it is plausible that declines in estradiol after menopause could increase bile acid abundances and influence CRC growth, particularly in females that have high cholesterol. In addition, bile acids have been linked to modulation of the immune response to cancer [27, 28], therefore bile acid abundances could affect the prognosis of patients with CRC [29, 30].

We observed that tissue abundances of most bile acids in CRC patients aged ≥ 55 years were higher in RCCs than in LCCs, independent of sex. In a separate CRC patient cohort from TCGA, we also observed that BSH-expressing bacteria, were more abundant on RCCs compared to LCCs. Sex-specific differences were observed when comparing RCC bile acid abundances to LCCs within each sex, wherein the secondary bile acids (DCA, LCA, and UDCA) were only increased in RCCs males. In females, these bile acids were at similar abundances in both RCCs and LCCs. Additional sex differences in conjugated bile acids (GCA, TDCA, GDCA, and GUDCA) were seen in stage II patients. Of note, no significant sex-specific differences were observed for patients < 55 years old, which could be due to the smaller sample size. However, RCCs from these younger patients show a trend of higher abundance of bile acids compared to LCC in males, and this trend was not seen in females. Since premenopausal females have much higher estradiol abundances than age-matched males, a plausible explanation for this sex-related difference is that estradiol lowers cholesterol and bile acid secretion, thus lower abundances are seen in the right colon of females. The finding of sex-specific differences in conjugated bile acids is of interest since conjugated bile acids function not only to facilitate lipid absorption but also to modulate bacterial growth in the intestine [31–33]. The enzymatic reaction catalyzed by BSH from resident microbiota has been recognized as the gateway in metabolism of conjugated bile acids [34]. BSH hydrolyzes the amino acids (taurine or glycine) from the sterol core of the conjugated bile acids. Despite the fact that conjugated primary bile acids are major substrates for BSH, members of microbiota with BSH activity are also able to act on conjugated secondary bile acids to liberate secondary bile acids [24]. A future study focused on microbial analysis of stool or tissues from CRC patients would be of importance to examine whether microbiome genera account for the differences in bile acid profiles between females and males. Another factor that may contribute to the sex-specific differences observed is possible differences in bile acid transporters that exist in the intestine and colon. The hepatic uptake of bile acids is mediated by Na^+^-TCA cotransporting polypeptide (NTCP) and Na^+^-independent transport by organic anion-transporting polypeptides (OATPs). A recent study showed that glycine and taurine-conjugated bile acids are preferable substrates for OATP1B1 and OATP1B3, however the sex-related expression differences in these proteins in humans are not known [35].

This study also identified that the ratio of GUDCA to UDCA and GCDCA to CDCA was significantly associated with shortened 5-year OS and RFS, respectively, in patients aged ≥ 55 years old. Multiple epidemiological studies have indicated that specific bile acids present in feces and blood samples are positively related to increased CRC risk, and one recent study revealed a correlation between bile acids and CRC outcomes [36–40], however these observations have not been consistently observed [38]. A recent prospective study showed that the prediagnostic abundance of GCDCA, in the serum is positively associated with risk of colon cancer [37]. In another study using serum samples from the Prostate, Lung, Colorectal and Ovarian Cancer Screening Trial (*n* = 254 colon cancer cases, *n* = 254 controls), serum GCDCA was the most strongly associated metabolite with CRC risk (out of 636 analyzed) among females (OR = 5.34), and similarly GUDCA was also significantly associated with increased risk (OR = 3.15) [41]. These associations did not change after adjustment for history of gallbladder disease or hormone therapy use. The bile acids were not significantly associated with males in the PLCO cohort [41]. Due to a limited number of deaths events compared to number of patients in each subgroup by sex our models did not give reliable estimates to determine the association of GCDCA, GUDCA, and their ratios to the unconjugated forms with outcomes. However, our data does show that female patients with RCC have higher levels of glycine-conjugated bile acids compared to LCCs, whereas there are no differences in these conjugates by side for males. Therefore, this sex-specificity would be of importance to investigate further. UDCA has been associated with decreased risk of colon cancer through its actions on increasing the hydrophilicity and decreasing the hydrophobicity of the bile acid pool. As GUDCA is a precursor for UDCA, we hypothesize that decreased deconjugation of GUDCA to UDCA could reduce its bioavailability in the colon. Although the mechanisms of glycine-conjugated bile acids and poorer survival remains unknown, it is plausible that reduced bio-transformation by the gut microbiome prevents the clearance of glycine-conjugated bile acids. Alternatively, increased production of glycine conjugates could be due to dietary or other underlying reasons, which may link to mechanisms that lead to increased recurrence and poorer overall survival. Another potential mechanism is the action of these glycine-conjugated bile acids on various receptors, in rodents, glycine-conjugated bile acids (GCA, GDCA and GCDCA) have been shown to decrease Farnesoid X Receptor (FXR) expression, which is required for maintaining the intestinal barrier, FXR loss has been associated with increased cancer [42]. In addition, GCA and GCDCA have been shown to stimulate the sphingosine-1-phosphate receptor 2 (S1PR2) in the rodent liver, and GDCA can stimulate S1PR2 in the intestine resulting in activation of extracellular regulated kinase (ERK)1/2 and AKT pathways [43]. Therefore, glycine-conjugated bile acids can have many roles in cell signaling, however it is not clear if these findings are translatable into humans. Most studies have focused on the unconjugated secondary bile acids DCA and LCA, in terms of tumor initiation or promotion [38, 44]. However, the high ratios of GUDCA and GCDCA to their deconjugated counterparts could signify the importance of these metabolites in CRC outcomes.

Increasing evidence shows that bile acids modulate immune responses in cancer. In this study, we found that the ratio of GUDCA to UDCA in both RCC and LCC colon tumors was positively correlated with FoxP3 + Treg cell levels but was not significantly correlated with CD8 + T cells. In addition, GUDCA and GCDCA were positively correlated with FoxP3 + Treg cell levels. In CRC Tregs are required to maintain intestinal immune tolerance, and the microbial environment promotes Treg differentiation. Studies have shown that FoxP3 Tregs associate with poorer OS, and potentially creates an immunosuppressive microenvironment [45, 46]. In animal models it is possible that the bile acids may be linked directly or indirectly to immune responses whereby the gut microbiome can mediate the population and activity of immune cells through bile acids [19, 47]. Thus, it is plausible that the microbiome could be responsible in part for the correlations observed between glycine-conjugates and immune cells in colon cancer.

### Perspectives and significance

In this study, we examined bile acid abundances in tumors from a large patient cohort, and observed differences by tumor location, sex, immune cell levels, and patient prognosis. Our study suggests that colon cancer patient outcomes could be linked to dampened immune responses that correlate with glycine-conjugated bile acid abundance. Although our study suggests that manipulation of bile acid abundances can benefit clinical care colon cancer patients, future studies are needed on the consideration of tumor location and sex of colon cancer patients towards precision medicine.

## Methods

### Chemicals and reagents

Cholic acid (CA), chenodeoxycholic acid (CDCA), sodium glycochenodeoxycholate (GCDCA), sodium taurochenodeoxycholate (TCDCA), glycocholic acid (GCA), taurolithocholic acid (TLCA), sodium taurodeoxycholate hydrate (TDCA), and taurocholic acid sodium salt hydrate (TCA) were purchased from Sigma-Aldrich (Saint Louis, MI). Ursodeoxycholic acid (UDCA) and deoxycholic acid (DCA) were purchased from ChemCruz (Santa Cruz Biotechnology, Inc., Dallas TX). Lithocholic acid (LCA) and glycoursodeoxycholic acid (GUDCA) were purchased from Cayman Chemical Company, (Michigan, USA). Glycodeoxycholic acid (GDCA) and glycolithocholic acid (GLCA) were purchased from Toronto Research Chemicals (TRC, Canada). Formic acid (99 + %) was purchased in 1 mL ampules from Thermo Scientific (Rockford, IL, USA). Ammonium formate and 2-propanol (both Optima® LC/MS grade), acetonitrile, methanol and water (both Optima® grade) were purchased from Fisher Chemical (Fair Lawn, NJ, USA).

### Sample collection

Colon tumors and normal colon tissues were acquired prospectively from 736 stage I–IV CRC patients during the period 1991–2001 at Memorial Sloan-Kettering Cancer Center (MSKCC, New York, NY, United States). Clinical data were recorded and updated retrospectively. Tumor tissue and normal colon tissue (away from the tumor at the resection margin) was acquired from surgical colectomy specimens. Each sample was snap frozen in liquid nitrogen and immediately stored in a -80 °C freezer. Pre-operative intravenous antibiotics (cefazolin/metronidazole, clindamycin/gentamicin or ciprofloxacin/metronidazole) were administered within 60 min prior to resection. All patients received a standard mechanical bowel preparation (polyethylene glycol (PEG) solution) 24 h before scheduled surgery. For this study, tumor tissue samples were selected from all RCCs and LCCs stage I–III patients (*n* = 228). Stage IV tumor samples were not included as their metabolism may be affected by the presence of metastases in the liver or other site, and were also treat with chemotherapeutics before surgery, therefore we cannot rule out these confounders. The Yale University IRB determined that the study conducted in this publication was not considered Human Subjects Research and did not require IRB review (IRB/HSC# 1612018746). The study does not obtain data through intervention or interaction with the individual or does not use or obtain identifiable private information. Informed consent was waived as part of the study exemption.

### Tissue bile acid extraction

50 ± 1 mg of each tissue was homogenized in using 500 μL of UPLC-grade H_2_O. A Cryolys Evolution homogenizer (Bertin Corporation, Rockville, MD, United States) was used with 2 mL lysing tube (Bertin Corporation) and 1.4 mm ceramic zirconium oxide beads (Bertin Corporation) to homogenize the tissues. Each sample was processed six times for 20 s, at 6000 rpm with 5 s intervals. Dry ice was used to keep the temperature < 10 °C during homogenization. From the homogenized solution, 100 µL was taken and added to 1.5 mL polypropylene microcentrifuge tubes for subsequent metabolite extraction. A volume of 300 μL ice cold methanol was added to each sample as the extraction solvent. The samples were vortexed for 30 s, and sonicated for 10 min. To precipitate proteins, the samples were incubated for 2 h at − 20 °C, followed by centrifugation at 13,000 rpm (15,000 *g*) and 4 °C for 15 min. The resulting supernatant was removed and evaporated to dryness for 12 h using a vacuum concentrator (Thermo Scientific, Waltham, MA, USA). The dry extracts were then reconstituted in 100 µL of ACN:H_2_O (1:1, v/v), sonicated for 10 min, and centrifuged at 13,000 rpm (15,000 g) and 4 °C for 15 min to remove insoluble debris. The supernatant was transferred to UPLC autosampler vials (Thermo Scientific, MA, USA). A pooled quality control sample was prepared by mixing 5 μL of extracted solution from each sample into a UPLC autosampler vial. All the vials were capped and stored at − 80 °C prior to UPLC–MS analysis.

### LC–MS for bile acids measurement

A UPLC system (H-Class ACQUITY, Waters Corporation, MA, United States) coupled to a quadrupole time-of flight (QTOF) mass spectrometer (Xevo G2-XS QTOF, Waters Corporation, MA, United States) was used for MS data acquisition. A Waters ACQUITY UPLC BEH C18 column (particle size, 1.7 μm; 50 mm (length) × 2.1 mm (i.d.)) equipped with a BEH C18 VanGuard pre-column (5 × 2.1 mm, i.d.; 1.7 μm) was used for the UPLC-based separation of bile acids. The mobile phase consisted of A: aqueous buffer containing 1 mM ammonium formate and formic acid (pH 4.39) and B: acetonitrile/isopropanol (1:1 v/v) at a total flow rate of 0.4 mL/min. The linear gradient elution started at a ramp of 20–30% B (0–3 min), 30–40% B (3–4 min), 50–70% B (4–5 min), 70–90% B (5–5.2 min), continuing at 90% B up to 6 min. Then, 90–20% B in 0.1 min with 1.9 min equilibration time, for a total of 8 min. The injection volume for all samples and standard solutions was 2 μL. The column temperature was set at 55 °C.

For MS analysis, an electrospray ionization source was operated in negative mode (ESI −). The parameters were as follow: spray voltage 2 kV, cone voltage 30 V, source temperature 120 °C, desolvation temperature 500 °C, cone gas flow 50 L/h, desolvation gas flow 900 L/h. MassLynx 4.1.0 software was used to acquire the data (Waters Corporation, Milford, MA, USA). ToF-multiple reaction monitoring (MRM) mode was used to quantify bile acids with target enhancement, in which a precursor ion is selected by the quadrupole and fragmented in the collision cell. The ToF pusher is synchronized with the mass-to-charge ratio (m/z) of the precursor or a product ion, maximizing the duty cycle for a target m/z range and effecting an increase in response and selectivity. The MRM transitions (m/z Da) for the precursor ion and the product ions for the bile acids, as well as the retention time are listed in Additional file 1: Table S7. TargetLynx (Waters Corp., Milford, MA, USA) was used to integrate chromatograms of bile acids.

### Differential abundance of gut microbiome in CRC

RNA-Seq raw reads in FASTA format were downloaded from a TCGA CRC dataset at the Genomic Data Commons (GDC) data portal (https://portal.gdc.cancer.gov). The reads were then applied to analyze intra-tumor microbiome using Kraken TCGA microbial detection as previously described elsewhere[23, 48]. Briefly, after the quality check and cleaning, the pre-processed sequencing reads were mapped to human reference genomes for human transcript identification. The unmapped sequences were then aligned against all known bacterial and archaeal genomes using the ultrafast Kraken algorithm [49] with a default setting of 31-mers window search for taxonomic identification. With the removal of batch effects, the taxonomic count data were normalized into log-count per million (log-cpm) using the Voom algorithm followed by supervised normalization (SNM). The differential genus abundance (in log2 fold-change) between RCC and LCC was then determined using LIMMA package. The multiple comparisons were corrected using the FDR approach.

### Quantitative immunofluorescence (QIF) experiment

Frozen tissue samples (*n* = 9 tumors *n* = 1 normal tissue) were placed in cassette individually and were submerged in 10% neutral buffered formalin. Tissues remained in formalin solution for four hours, then each cassette was transferred to another container of 70% ethanol. The formalin-fixed tissues were submitted to the Yale Specialized Translational Services Laboratory (STS Lab) Core to examine expression of CD8 + , and CD4 + FoxP3 + . Antibodies were titrated using a 5 point titration model as previously described. Antibodies were validated using known positive controls of FFPE fixed human tissue and cell lines [50–52]. Antibodies shown to be selective in this process were used for QIF. The following antibodies were used to analyze protein expression in the colon tumor tissues: CD4 + (SP35 Abcam, Boston, MA), CD8 + (C8/144B Abcam), and FoxP3 + (D2W8E, Cell Signaling Technologies, Danvers, MA). The QIF automated quantitative staining experiment was conducted using standard protocols [53]. Analysis was carried out using Vectra Polaris Imaging System, Phenochart 1.0.10, InForm 2.4.8 and PhenoptrReports. Briefly, slides were imaged using the Vectra Polaris system, areas of interest (as determined by H + E) were annotated after a 4 × scan using Phenochart. FOV (field of view) defined in Phenochart were acquired as mutltispectral MSI and analyzed using tissue and cell segmentation and single cell type phenotyping in InForm. The number of FOVs examined for each tissue averaged 16 between the tissues. Each FOV was taken at 20 × resolution using the Akoya Phenoimager HT. Quantification and Phenotying were performed using InForm software (version 2.3). Briefly representative images for each sample (minimum 3 FOV per sample) were selected. Images were spectrally unmixed and trained for tissue and cell segmentation. Finally, cells were phenotyped as CK + , CD8 + , CD4 + Or Foxp3 + and others. Each + phenotype was done independently, and double positive phenotypes were assessed using R studio (V 1.1.463) and Phenoptyr reports (Akoya V1.1 Cell segmentation files were combined to detect double positives for each phenotype using PhenoptrReports. H&E staining was performed using standard protocols. QIF experiment was carried out by a facility staff member that was blinded to the patient sex and tumor location.

### Statistical analysis of bile acids data

Nonparametric Wilcoxon Mann–Whitney *U* test was used to find bile acids exhibiting significant differences between RCCs and LCCs. *p* values were adjusted for multiple testing with Benjamini–Hochberg-based FDR. The statistical analyses were performed using R (version 3.4.3).

### Association analysis of bile acids and overall survival and recurrence

According to the median value of each bile acid abundance among colon cancer patients, categorical variables low and high were reassigned. Kendall rank correlation coefficient were calculated to evaluate correlations between bile acids using function “cor” in R (version 3.4.3). Multivariable Cox proportional hazard (PH) regression models with hazard ratios and 95% confidence intervals were constructed using package “survival” in R (version 3.4.3). Due to insufficient numbers of death events in female patients within clinical stage I, we counted patients within clinical stage I and II together as “early stage”, while patients of clinical stage III were coded as “late stage”. Variables in the model obey the proportional hazard assumption. The assumption of proportional hazard was checked using R function “cox.zph”. The R code is available in Additional file 3: Additional data, and a spreadsheet combining clinical information and bile acid values (dichotomized by medians) is shown in Additional File 4: Table S8.

### Statistical analysis of QIF data

Pearson correlation analyses between abundance of each individual immune cell and metabolite abundance/ratio were performed using package “ggpubr” in R (version 3.4.3). The correlation coefficient and *p* value were calculated.

## Supplementary Information

**Additional file 1: Table S1**. Names of the bile acids measured in tumor tissues. **Table S2**. Demographics of colon cancer patients from samples used within this study. **Table S4. **Comparison of bile acid abundance between RCCs and LCCs by sex and age. **Table S5.** Pearson correlation of glucuronic acid to bile acid abundances in patients with LCC ≥ 55 years old (n=99). **Table S6.** Correlations between prognosis-linked bile acid and T regulatory cell abundances in RCC tissues (n=6). **Table S7. **Multiple reaction monitoring transitions, retention times and collision energy levels for the detection of bile acids in colon tumor tissues. **Fig. S1.** Bile acid abundances in colon tumors from patients < 55 years old. Bile acid abundance measured in stage I–III tumors combined from left-sided colon cancers (LCCs, n = 14) and right-sided colon cancers (RCCs, n = 17) from patients with age < 55 years old. Data represent median with interquartile range. Nonparametric Wilcoxon Mann–Whitney U test, *p* values adjusted for false discovery rates (FDR) (Benjamini–Hochberg). ns. = not significant. CA, cholic acid; CDCA, chenodeoxycholic acid; TCA, taurocholic acid; TCDCA, taurochenodeoxycholic acid; GCA, glycocholic acid; GCDCA, glycochenodeoxycholic acid; DCA, deoxycholic acid; LCA, lithocholic acid; UDCA, ursodeoxycholic acid; TDCA, taurodeoxycholic acid; TLCA, taurolithocholic acid; GDCA, glycodeoxycholic acid; GLCA, glycolithocholic acid; GUDCA, glycoursodeoxycholic acid. **Fig. S2**. Differences in tumor tissue bile acids between left-sided colon cancers (LCCs) and right-sided colon cancers (RCCs) from patients ≥ 55 years old by stage. (A) stages I (LCC n = 25, RCC n = 22), and (B) stage II (LCC n = 42, RCC n = 44), and (C) stage III (LCCs, n = 32, RCCs, n = 32). Data represent median with interquartile range. Nonparametric Wilcoxon Mann–Whitney U test, *p* values adjusted for false discovery rates (FDR) (Benjamini–Hochberg). ns. = not significant. CA, cholic acid; CDCA, chenodeoxycholic acid; TCA, taurocholic acid; TCDCA, taurochenodeoxycholic acid; GCA, glycocholic acid; GCDCA, glycochenodeoxycholic acid; DCA, deoxycholic acid; LCA, lithocholic acid; UDCA, ursodeoxycholic acid; TDCA, taurodeoxycholic acid; TLCA, taurolithocholic acid; GDCA, glycodeoxycholic acid; GLCA, glycolithocholic acid; GUDCA, glycoursodeoxycholic acid. **Fig. S3.** Microbiota that differ in abundance between LCC and RCC tumor tissues using data from TCGA COAD. Abundances (log2) of A) *Faecalibacterium,* B) *Coprococcus*, C) *Dorea*, D) *Luteibacter,* and E) *Sorangium*, differences determined by t-test *FDR corrected p<0.05, RCC, n=136; LCC, n=262. **Fig. S4**. No differences in tumor tissue bile acids between tumors from male and female patients ≥ 55 years old. (A) left-sided colon cancers (LCCs) and right-sided colon cancers (RCCs) combined, male n=118, female n=110 (B) RCCs, male n = 55, female n = 60, and (C) LCCs, male n = 63, female n = 50. Data represent median with interquartile range. Nonparametric Wilcoxon Mann–Whitney U test, *p* values adjusted for false discovery rates (FDR) (Benjamini–Hochberg) to compare bile acids between male and female patients, all comparisons were not statistically significant. CA, cholic acid; CDCA, chenodeoxycholic acid; TCA, taurocholic acid; TCDCA, taurochenodeoxycholic acid; GCA, glycocholic acid; GCDCA, glycochenodeoxycholic acid; DCA, deoxycholic acid; LCA, lithocholic acid; UDCA, ursodeoxycholic acid; TDCA, taurodeoxycholic acid; TLCA, taurolithocholic acid; GDCA, glycodeoxycholic acid; GLCA, glycolithocholic acid; GUDCA, glycoursodeoxycholic acid. **Fig. S5**. Kendall correlations between bile acids among patients with age ≥ 55 years old (n=197). Box with numerical value suggest that the correlation coefficient is calculated as equal or larger than 0.8. CA, cholic acid; CDCA, chenodeoxycholic acid; TCA, taurocholic acid; TCDCA, taurochenodeoxycholic acid; GCA, glycocholic acid; GCDCA, glycochenodeoxycholic acid; DCA, deoxycholic acid; LCA, lithocholic acid; UDCA, ursodeoxycholic acid; TDCA, taurodeoxycholic acid; TLCA, taurolithocholic acid; GDCA, glycodeoxycholic acid; GLCA, glycolithocholic acid; GUDCA, glycoursodeoxycholic acid. **Fig. S6**. Linear regression of CD4+T cell abundances examined by quantitative immunofluorescence (QIF), against the ratios of GUDCA/UDCA. All patients with CRC (n=10) and patients with RCC (n=6) * P<0.05, 95% confidence bands shown.

**Additional file 2: Table S3.** Statistics of the abundances of bacteria in tumors from RCCs and LCCs.

**Additional file 3.** R code for performing survival analysis.

**Additional file 4.** Clinical information and bile acid values (dichotomized by medians) of individual patients.

## Data Availability

The raw data generated during the current study are available from the corresponding author on reasonable request. The processed bile acid data and microbiome data used in the figures presented in the manuscript are supplied as Supplementary Data.

## Abbreviations

CRC: Colorectal cancer
RCC: Right-sided colon cancer
LCC: Left-sided colon cancer
SEER: Surveillance, Epidemiology, and End Results
CDCA: Chenodeoxycholic acid
TCA: Taurocholic acid
LCA: Lithocholic acid
CA: Cholic acid
GCA: Glycocholic acid
TCDCA: Taurochenodeoxycholic acid
GCDCA: Glycochenodeoxycholic acid
DCA: Deoxycholic acid
UDCA: Ursodeoxycholic acid
TDCA: Taurodeoxycholic acid
GDCA: Glycodeoxycholic acid
GUDCA: Glycoursodeoxycholic acid
TLCA: Taurolithocholic acid
GLCA: Glycolithocholic acid
BSH: Bile salt hydrolase
PH: Proportional hazard
OS: Overall survival
RFS: Recurrence-free survival
QIF: Quantitative immunofluorescence
FXR: Farnesoid X Receptor
