# Supplementary material for: Bile acid distributions, sex-specificity, and prognosis in colorectal cancer
Source: Biol Sex Differ. 2022 Oct 23;13:61. doi: 10.1186/s13293-022-00473-9 (PMC9590160; doi:10.1186/s13293-022-00473-9)
Supplement: Supplementary file 1 — Additional file 1: Table S1. Names of the bile acids measured in tumor tissues. Table S2. Demographics of colon cancer patients from samples used within this study. Table S4. Comparison of bile acid abundance between RCCs and LCCs by sex and age. Table S5. Pearson correlation of glucuronic acid to bile acid abundances in patients with LCC ≥ 55 years old (n=99). Table S6. Correlations between prognosis-linked bile acid and T regulatory cell abundances in RCC tissues (n=6). Table S7. Multiple reaction monitoring transitions, retention times and collision energy levels for the detection of bile acids in colon tumor tissues. Fig. S1. Bile acid abundances in colon tumors from patients < 55 years old. Bile acid abundance measured in stage I–III tumors combined from left-sided colon cancers (LCCs, n = 14) and right-sided colon cancers (RCCs, n = 17) from patients with age < 55 years old. Data represent median with interquartile range. Nonparametric Wilcoxon Mann–Whitney U test, p values adjusted for false discovery rates (FDR) (Benjamini–Hochberg). ns. = not significant. CA, cholic acid; CDCA, chenodeoxycholic acid; TCA, taurocholic acid; TCDCA, taurochenodeoxycholic acid; GCA, glycocholic acid; GCDCA, glycochenodeoxycholic acid; DCA, deoxycholic acid; LCA, lithocholic acid; UDCA, ursodeoxycholic acid; TDCA, taurodeoxycholic acid; TLCA, taurolithocholic acid; GDCA, glycodeoxycholic acid; GLCA, glycolithocholic acid; GUDCA, glycoursodeoxycholic acid. Fig. S2. Differences in tumor tissue bile acids between left-sided colon cancers (LCCs) and right-sided colon cancers (RCCs) from patients ≥ 55 years old by stage. (A) stages I (LCC n = 25, RCC n = 22), and (B) stage II (LCC n = 42, RCC n = 44), and (C) stage III (LCCs, n = 32, RCCs, n = 32). Data represent median with interquartile range. Nonparametric Wilcoxon Mann–Whitney U test, p values adjusted for false discovery rates (FDR) (Benjamini–Hochberg). ns. = not significant. CA, cholic acid; CDCA, chenodeoxycholic acid; TCA, t [file 13293_2022_473_MOESM1_ESM.docx]

**Additional file 1: table S1**. Names of the bile acids measured in tumor tissues.

| Bile acid name and type | Abbreviation |  |
| --- | --- | --- |
|  | | |
| ***Primary unconjugated*** | | |
| Cholic acid | CA |  |
| Chenodeoxycholic acid | CDCA |  |
|  |  |  |
| ***Primary conjugated*** | | |
| Taurocholic acid | TCA |  |
| Taurochenodeoxycholic acid | TCDCA |  |
| Glycocholic acid | GCA |  |
| Glycochenodeoxycholic acid | GCDCA |  |
|  |  |  |
| ***Secondary unconjugated*** | | |
| Deoxycholic acid | DCA |  |
| Lithocholic acid | LCA |  |
| Ursodeoxycholic acid | UDCA |  |
|  |  |  |
| ***Secondary conjugated*** | | |
| Taurodeoxycholic acid | TDCA |  |
| Taurolithocholic acid | TLCA |  |
| Glycodeoxycholic acid | GDCA |  |
| Glycolithocholic acid | GLCA |  |
| Glycoursodeoxycholic acid | GUDCA |  |

**table S2**. Demographics of colon cancer patients from samples used within this study.

|  | **Stage I** (n=52) | | | **Stage II** (n=102) | | | **Stage III** (n=74) | | | | |
| --- | --- | --- | --- | --- | --- | --- | --- | --- | --- | --- | --- |
|  | **RCC**  (n=25) | | **LCC**  (n=27) |  | **RCC**  (n=54) | **LCC**  (n=48) | |  | **RCC**  (n=36) | **LCC**  (n=38) |  |
| **Sex, n** | |  |  |  |  |  | |  |  |  |  |
| Male | | 11 | 16 |  | 27 | 29 | |  | 17 | 18 |  |
| Female | | 14 | 11 |  | 27 | 19 | |  | 19 | 20 |  |
| **Age, years, n** | |  |  |  |  |  | |  |  |  |  |
| < 55 | | 3 | 2 |  | 10 | 6 | |  | 4 | 6 |  |
| ≥ 55 | | 22 | 25 |  | 44 | 42 | |  | 32 | 32 |  |
| **KRAS mutation, n** | | | | | | | | | | | |
| Wild type | | 7 | 15 |  | 29 | 26 | |  | 17 | 24 |  |
| Mutant | | 9 | 8 |  | 14 | 14 | |  | 12 | 10 |  |
| Unknown | | 9 | 4 |  | 11 | 8 | |  | 7 | 4 |  |
| **BRAF mutation, n** | | | | | | | | | | | |
| Wild type | | 14 | 20 |  | 27 | 36 | |  | 21 | 33 |  |
| Mutant | | 0 | 0 |  | 9 | 1 | |  | 8 | 0 |  |
| Unknown | | 11 | 7 |  | 18 | 11 | |  | 7 | 5 |  |
| **Microsatellite instability** **status, n** | | | | | | | | | | | |
| MSS | | 10 | 17 |  | 20 | 30 | |  | 18 | 23 |  |
| MSI | | 6 | 4 |  | 21 | 7 | |  | 10 | 8 |  |
| Unknown | | 9 | 6 |  | 13 | 11 | |  | 8 | 7 |  |
| **Number of positive lymph nodes, n** | | | | | | | | | | | |
| 0 | | 24 | 27 |  | 53 | 48 | |  | 0 | 0 |  |
| 1-2 | | 0 | 0 |  | 0 | 0 | |  | 26 | 31 |  |
| ≥3 | | 1 | 0 |  | 1 | 0 | |  | 10 | 7 |  |
| **Race and Ethnicity, n** | | | | | | | | | | | |
| White | | 21 | 23 |  | 50 | 41 | |  | 29 | 29 |  |
| Others | | 4 | 4 |  | 4 | 7 | |  | 7 | 9 |  |
| **Received preoperative chemotherapy, n** | | | | | | | | | | | |
| Yes | | 1 | 0 |  | 9 | 9 | |  | 30 | 30 |  |
| No | | 24 | 27 |  | 45 | 39 | |  | 6 | 8 |  |

RCC, right-sided colon cancer; LCC, left-sided colon cancer; MSI, microsatellite instable; MSS, microsatellite stable

**table S4.** Comparison of bile acid abundance between RCCs and LCCs by sex and age.

|  |  | Age ≥ 55 years, Stages I-III | | | | Age < 55 years, Stages I-III | | | | |
| --- | --- | --- | --- | --- | --- | --- | --- | --- | --- | --- |
|  |  | Female  (RCC vs LCC) | | Male  (RCC vs LCC) | | Female  (RCC vs LCC) | | | Male  (RCC vs LCC) | |
|  |  | FC ^a^ | P value ^b^ | FC ^a^ | P value ^b^ | FC ^a^ | P value ^b^ | FC ^a^ | | P value ^b^ |
| **Primary BAs** | CA | 4.4 | 0.039 | 4.6 | 0.004 | 0.1 | ns. | 0.5 | | ns. |
|  | CDCA | 2.7 | 0.034 | 4.7 | 0.001 | 2.4 | ns. | 3.4 | | ns. |
| Taurine-conjugated primary BAs | TCA | 15.2 | 0.011 | 6.5 | 0.004 | 0.4 | ns. | 6.9 | | ns. |
|  | TCDCA | 8.6 | 0.003 | 8.4 | 0.002 | 2.4 | ns. | 16.7 | | ns. |
| Glycine-conjugated primary BAs | GCA | 12.0 | 0.022 | 9.8 | 0.005 | 0.03 | ns. | 7.7 | | ns. |
|  | GCDCA | 10.4 | 0.022 | 11.2 | 0.002 | 0.04 | ns. | 8.3 | | ns. |
| **Secondary BAs** | DCA* | 1.4 | ns. | 3.2 | 0.001 | 0.8 | ns. | 1.9 | | ns. |
|  | LCA* | 1.2 | ns. | 1.4 | 0.002 | 1.1 | ns. | 1.2 | | ns. |
|  | UDCA* | 1.5 | ns. | 3.8 | 0.001 | 2.8 | ns. | 4.6 | | ns. |
| Taurine-conjugated secondary BAs | TDCA | 2.4 | 0.031 | 3.1 | 0.038 | 0.5 | ns. | 7.7 | | ns. |
|  | TLCA | 1.1 | ns. | 1.2 | ns. | 0.8 | ns. | 2.9 | | ns. |
| Glycine-conjugated secondary BAs | GDCA | 7.7 | 0.031 | 3.2 | 0.050 | 0.03 | ns. | 49.5 | | ns. |
|  | GLCA | 1.5 | ns. | 2.2 | ns. | 0.04 | ns. | 5.3 | | ns. |
|  | GUDCA | 10.9 | 0.031 | 17.2 | 0.001 | 0.07 | ns. | 18.9 | | ns. |

^a^ FC=Fold change, fold change calculated by dividing median value of RCC by median value of LCC. ^b^ P-values estimated by the Mann-Whitney U test adjusted for false discovery rates (FDR) (Benjamini-Hochberg). ns. = not significant. ^*^ Bile acids identified with sex-specific differences.BA, bile acids; RCC, right-sided colon cancer; LCC, left-sided colon cancer; CA, cholic acid; CDCA, chenodeoxycholic acid; TCA, taurocholic acid; TCDCA, taurochenodeoxycholic acid; GCA, glycocholic acid; GCDCA, glycochenodeoxycholic acid; DCA, deoxycholic acid; LCA, lithocholic acid; UDCA, ursodeoxycholic acid; TDCA, taurodeoxycholic acid; TLCA, taurolithocholic acid; GDCA, glycodeoxycholic acid; GLCA, glycolithocholic acid; GUDCA, glycoursodeoxycholic acid.

**table S5.** Pearson correlation of glucuronic acid to bile acid abundances in patients with LCC ≥ 55 years old (n=99).

| Bile Acid | R | P value |
| --- | --- | --- |
| CA | 0.691 | <0.001 |
| CDCA | 0.206 | ns. |
| GCA | 0.301 | ns. |
| DCA | 0.261 | <0.001 |
| LCA | 0.278 | ns. |
| UDCA | 0.226 | ns. |
| TDCA | 0.450 | ns. |
| TLCA | 0.026 | <0.010 |
| GDCA | 0.164 | ns. |
| GLCA | 0.143 | <0.001 |
| TCA | 0.353 | <0.050 |
| TCDCA | 0.045 | ns. |
| GCA | 0.464 | <0.050 |
| GCDCA | 0.205 | ns. |

R = Pearson correlation coefficient. CA, cholic acid; CDCA, chenodeoxycholic acid; TCA, taurocholic acid; TCDCA, taurochenodeoxycholic acid; GCA, glycocholic acid; GCDCA, glycochenodeoxycholic acid; DCA, deoxycholic acid; LCA, lithocholic acid; UDCA, ursodeoxycholic acid; TDCA, taurodeoxycholic acid; TLCA, taurolithocholic acid; GDCA, glycodeoxycholic acid; GLCA, glycolithocholic acid; GUDCA, glycoursodeoxycholic acid.

**table S6.** Correlations between prognosis-linked bile acid and T regulatory cell abundances in RCC tissues (n=6).

| Bile Acid | CD8+ | | FoxP3+ | |
| --- | --- | --- | --- | --- |
|  | R | P value | R | P value |
| GUDCA | -0.31 | 0.560 | 0.86 | 0.028 |
| GCDCA | -0.18 | 0.720 | 0.92 | 0.009 |
| GCDCA/CDCA | -0.18 | 0.740 | 0.67 | 0.150 |
| GUDCA/UDCA | 0.08 | 0.870 | 0.77 | 0.070 |

**table S7.** Multiple reaction monitoring transitions, retention times and collision energy levels for the detection of bile acids in colon tumor tissues.

| Bile acid | Precursor ion  [M-H]^-^ | Product ion | Retention  time (min) | Collision energy  (CE, eV) |
| --- | --- | --- | --- | --- |
| CA | 407.2802 | 407.2802 | 4.40 | 0 |
| CDCA | 391.2853 | 391.2853 | 4.85 | 0 |
| TCA | 514.2843 | 79.9568 | 3.30 | -65 |
| TCDCA | 498.2894 | 79.9568 | 4.11 | -70 |
| GCA | 464.3017 | 74.0244 | 3.55 | -55 |
| GCDCA | 448.3068 | 74.0244 | 4.22 | -40 |
| DCA | 391.2853 | 391.2853 | 4.93 | 0 |
| LCA | 375.2904 | 375.2904 | 5.31 | 0 |
| UDCA | 391.2853 | 391.2853 | 4.38 | 0 |
| TDCA | 498.2894 | 79.9568 | 4.23 | -70 |
| TLCA | 482.2946 | 79.9568 | 4.57 | -70 |
| GDCA | 448.3068 | 74.0244 | 4.33 | -40 |
| GLCA | 432.3119 | 74.0244 | 4.69 | -40 |
| GUDCA | 448.3068 | 74.0244 | 3.22 | -40 |

CE, collision energy; CA, cholic acid; CDCA, chenodeoxycholic acid; TCA, taurocholic acid; TCDCA, taurochenodeoxycholic acid; GCA, glycocholic acid; GCDCA, glycochenodeoxycholic acid; DCA, deoxycholic acid; LCA, lithocholic acid; UDCA, ursodeoxycholic acid; TDCA, taurodeoxycholic acid; TLCA, taurolithocholic acid; GDCA, glycodeoxycholic acid; GLCA, glycolithocholic acid; GUDCA, glycoursodeoxycholic acid.


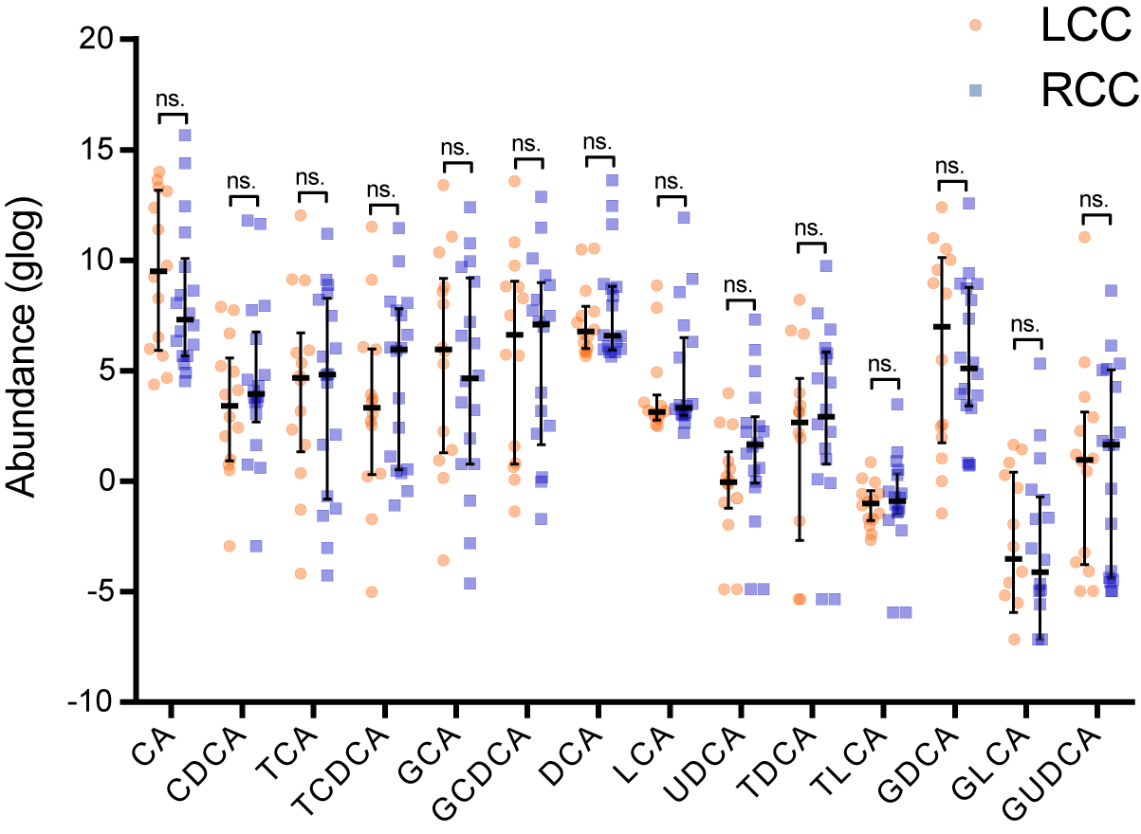


**Fig. S1.** **Bile acid abundances in colon tumors from patients < 55 years old.** Bile acid abundance measured in stage I-III tumors combined from left-sided colon cancers (LCCs, n = 14) and right-sided colon cancers (RCCs, n = 17) from patients with age < 55 years old. Data represent median with interquartile range. Nonparametric Wilcoxon Mann-Whitney U test, *p* values adjusted for false discovery rates (FDR) (Benjamini-Hochberg). ns. = not significant. CA, cholic acid; CDCA, chenodeoxycholic acid; TCA, taurocholic acid; TCDCA, taurochenodeoxycholic acid; GCA, glycocholic acid; GCDCA, glycochenodeoxycholic acid; DCA, deoxycholic acid; LCA, lithocholic acid; UDCA, ursodeoxycholic acid; TDCA, taurodeoxycholic acid; TLCA, taurolithocholic acid; GDCA, glycodeoxycholic acid; GLCA, glycolithocholic acid; GUDCA, glycoursodeoxycholic acid.

**
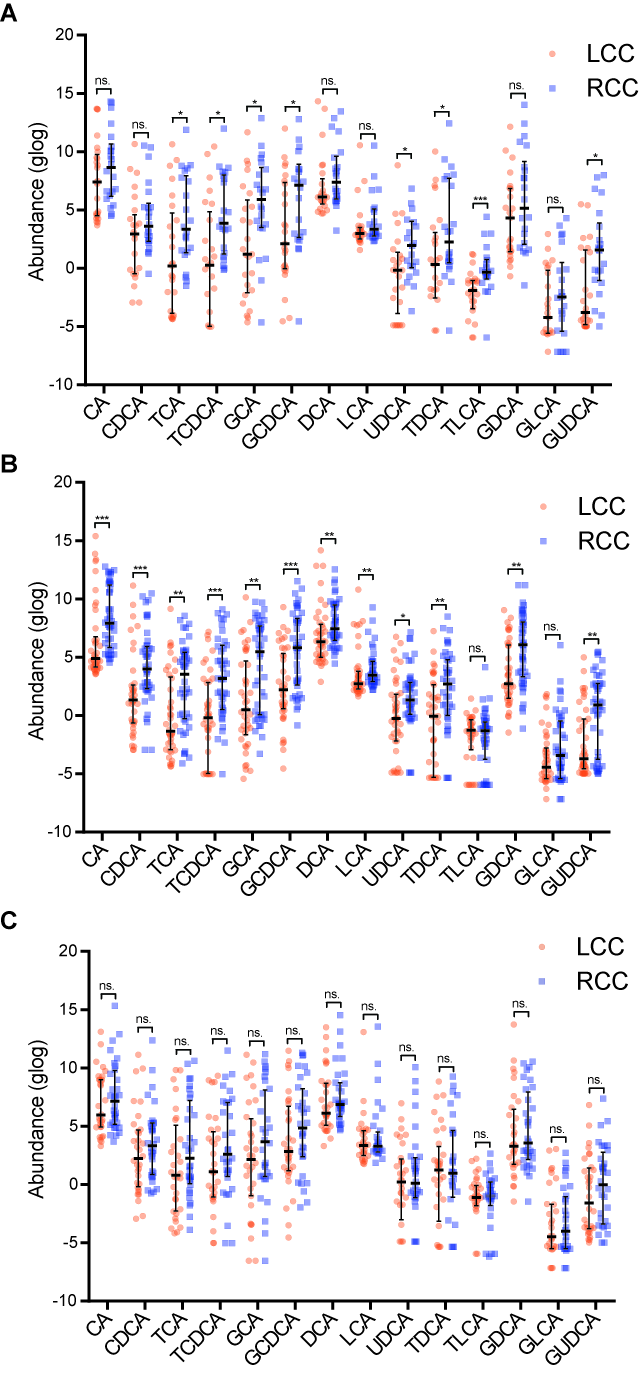
**

**Fig. S2**. **Differences in tumor tissue bile acids between left-sided colon cancers (LCCs) and right-sided colon cancers (RCCs) from patients ≥ 55 years old by stage.** (**A**) stages I (LCC n = 25, RCC n = 22), and **(B)** stage II (LCC n = 42, RCC n = 44), and **(C)** stage III (LCCs, n = 32, RCCs, n = 32). Data represent median with interquartile range. Nonparametric Wilcoxon Mann-Whitney U test, *p* values adjusted for false discovery rates (FDR) (Benjamini-Hochberg). ns. = not significant. CA, cholic acid; CDCA, chenodeoxycholic acid; TCA, taurocholic acid; TCDCA, taurochenodeoxycholic acid; GCA, glycocholic acid; GCDCA, glycochenodeoxycholic acid; DCA, deoxycholic acid; LCA, lithocholic acid; UDCA, ursodeoxycholic acid; TDCA, taurodeoxycholic acid; TLCA, taurolithocholic acid; GDCA, glycodeoxycholic acid; GLCA, glycolithocholic acid; GUDCA, glycoursodeoxycholic acid.


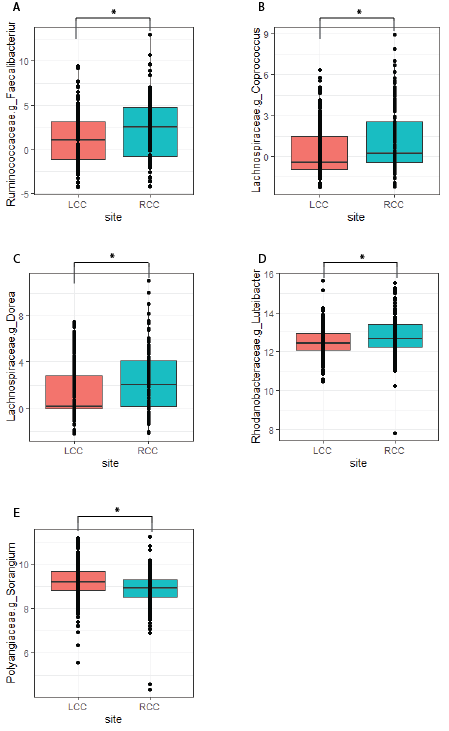


**Fig. S3. Microbiota that differ in abundance between LCC and RCC tumor tissues using data from TCGA COAD.** Abundances (log2) of A) *Faecalibacterium,* B) *Coprococcus,* C) *Dorea*, D) *Luteibacter,* and E) *Sorangium*, differences determined by t-test *FDR corrected p<0.05, RCC, n=136; LCC, n=262.

**
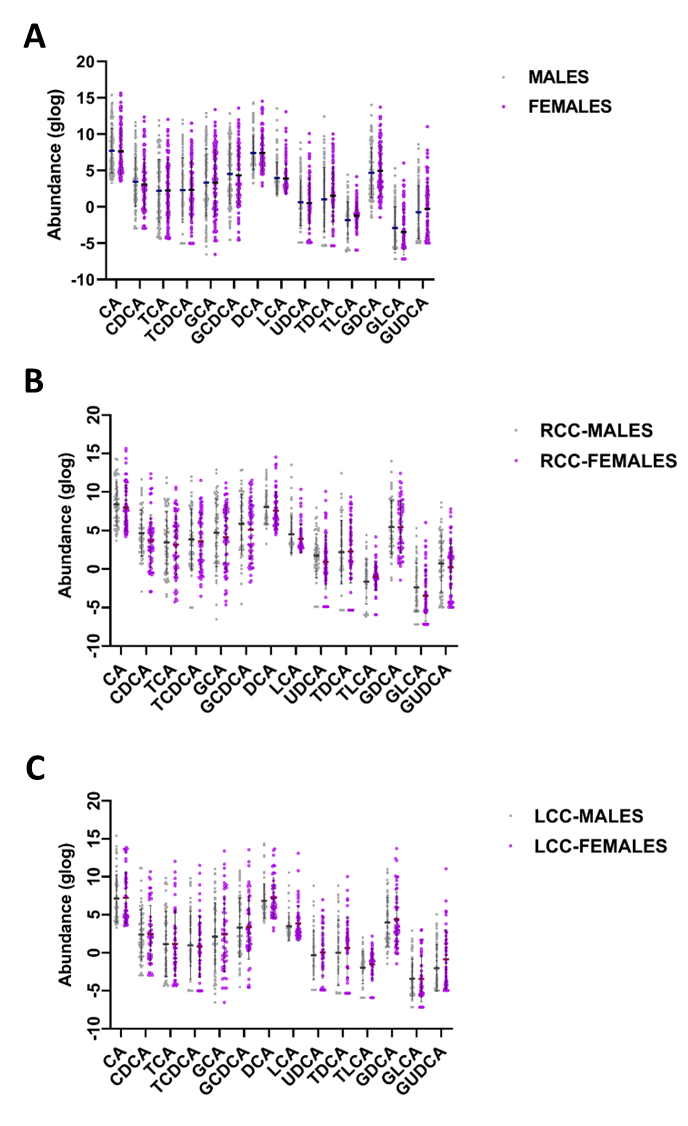
**

**Fig. S4**. **No differences in tumor tissue bile acids between tumors from male and female patients ≥ 55 years old.** (**A**) left-sided colon cancers (LCCs) and right-sided colon cancers (RCCs) combined, male n=118, female n=110 **(B)** RCCs, male n = 55, female n = 60, and **(C)** LCCs, male n = 63, female n = 50. Data represent median with interquartile range. Nonparametric Wilcoxon Mann-Whitney U test, *p* values adjusted for false discovery rates (FDR) (Benjamini-Hochberg) to compare bile acids between male and female patients, all comparisons were not statistically significant. CA, cholic acid; CDCA, chenodeoxycholic acid; TCA, taurocholic acid; TCDCA, taurochenodeoxycholic acid; GCA, glycocholic acid; GCDCA, glycochenodeoxycholic acid; DCA, deoxycholic acid; LCA, lithocholic acid; UDCA, ursodeoxycholic acid; TDCA, taurodeoxycholic acid; TLCA, taurolithocholic acid; GDCA, glycodeoxycholic acid; GLCA, glycolithocholic acid; GUDCA, glycoursodeoxycholic acid.


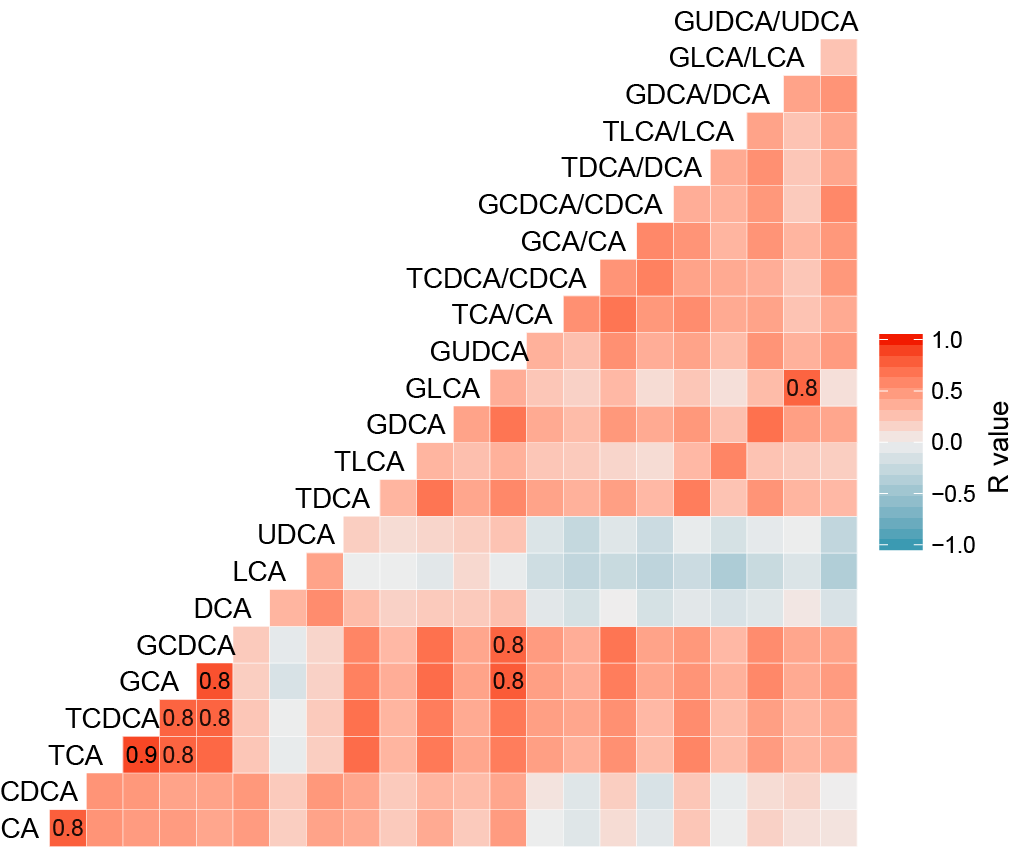


**Fig. S5**. **Kendall correlations between bile acids among patients with age ≥ 55 years old (n=197)**. Box with numerical value suggest that the correlation coefficient is calculated as equal or larger than 0.8. CA, cholic acid; CDCA, chenodeoxycholic acid; TCA, taurocholic acid; TCDCA, taurochenodeoxycholic acid; GCA, glycocholic acid; GCDCA, glycochenodeoxycholic acid; DCA, deoxycholic acid; LCA, lithocholic acid; UDCA, ursodeoxycholic acid; TDCA, taurodeoxycholic acid; TLCA, taurolithocholic acid; GDCA, glycodeoxycholic acid; GLCA, glycolithocholic acid; GUDCA, glycoursodeoxycholic acid.


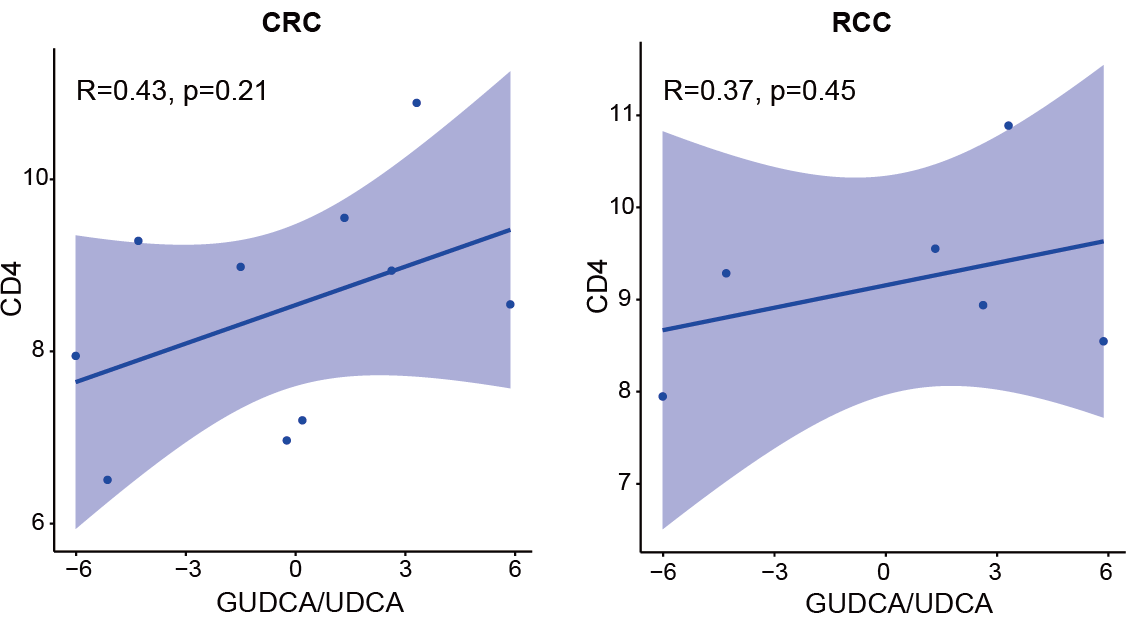


**Fig. S6**. **Linear regression of CD4+T cell abundances examined by quantitative immunofluorescence (QIF), against the ratios of GUDCA/UDCA.** All patients with CRC (n=10) and patients with RCC (n=6) * P<0.05, 95% confidence bands shown.
