# Supplementary material for: Bile acid distributions, sex-specificity, and prognosis in colorectal cancer
Source: Biol Sex Differ. 2022 Oct 23;13:61. doi: 10.1186/s13293-022-00473-9 (PMC9590160; doi:10.1186/s13293-022-00473-9)
Supplement: Supplementary file 3 — Additional file 3. R code for performing survival analysis. [file 13293_2022_473_MOESM3_ESM.docx]

**Additional File 3**

R code for performing survival analysis

library(survival)
setwd("Your working directory")
cox.os<-read.csv("CoxData_197samples_L55_lowhigh.csv")

cox.os$SexGroup<-ifelse(cox.os$SexGroup == "a", 'm', 'f') #"a" means male, "b" means female
cox.os$SideGroup<-ifelse(cox.os$SideGroup == "a", 'L', 'R') #"a" means LCC, "b" means RCC
cox.os$Chemo<-ifelse(cox.os$Chemo != "0", '1', '0') # 0 means no chemotherapy history
cox.os$stageGroup<-ifelse(cox.os$stageGroup == "a"|cox.os$stageGroup=="b", 'early', 'late') # "a" means stage I, "b" means stage II, "c" means stage III. stage I and II were further categorized as "early stage", and stage III refers to "late stage".
cox.os$stageGroup<-factor(cox.os$stageGroup)
cox.os$SexGroup<-factor(cox.os$SexGroup)
cox.os$SideGroup<-factor(cox.os$SideGroup)
cox.os$Chemo<-factor(cox.os$Chemo)

Sur1 <- Surv(time = cox.os$OS5.months, event = cox.os$OS5.status)
Sur2 <- Surv(time = cox.os$Recurrence5.months, event = cox.os$RecurrenceStatus)

MultiNames <- c("SexGroup", "SideGroup", "stageGroup", "age","Chemo", "CA", "GCA", "DCA", "LCA", "UDCA", "TDCA", "TLCA", "GDCA","TCA_CA", "TCDCA_CDCA", "GCA_CA", "GCDCA_CDCA", "TDCA_DCA", "TLCA_LCA", "GDCA_DCA", "GLCA_LCA", "GUDCA_UDCA")

#Overall Survival
FML <- as.formula(paste0('Sur1~',paste(MultiNames,collapse = '+')))
MultiMod <- coxph(FML,data = cox.os)
SMultiMod <- summary(MultiMod)
HR <- round(SMultiMod$coefficients[,2],2)
P_Value <- round(SMultiMod$coefficients[,5],3)
LCI <- round(SMultiMod$[conf.int](https://nam12.safelinks.protection.outlook.com/?url=http%3A%2F%2Fconf.int%2F&data=05%7C01%7Ccaroline.johnson%40yale.edu%7C4c483d99d8444b6f17de08da610f3a81%7Cdd8cbebb21394df8b4114e3e87abeb5c%7C0%7C0%7C637929015283336878%7CUnknown%7CTWFpbGZsb3d8eyJWIjoiMC4wLjAwMDAiLCJQIjoiV2luMzIiLCJBTiI6Ik1haWwiLCJXVCI6Mn0%3D%7C3000%7C%7C%7C&sdata=FIiYgQkxa293r7YkFqNxzxTEqBGZKBuSBwzmKf1IJao%3D&reserved=0)[,3],2)
UCI <- round(SMultiMod$[conf.int](https://nam12.safelinks.protection.outlook.com/?url=http%3A%2F%2Fconf.int%2F&data=05%7C01%7Ccaroline.johnson%40yale.edu%7C4c483d99d8444b6f17de08da610f3a81%7Cdd8cbebb21394df8b4114e3e87abeb5c%7C0%7C0%7C637929015283336878%7CUnknown%7CTWFpbGZsb3d8eyJWIjoiMC4wLjAwMDAiLCJQIjoiV2luMzIiLCJBTiI6Ik1haWwiLCJXVCI6Mn0%3D%7C3000%7C%7C%7C&sdata=FIiYgQkxa293r7YkFqNxzxTEqBGZKBuSBwzmKf1IJao%3D&reserved=0)[,4],2)
CI95 <- paste(LCI,'-',UCI)
Multi.OS <- data.frame(Characteristics = MultiNames[1:length(MultiNames)],
                    Hazard_Ratio = HR[1:length(MultiNames)],
                    CI95 = CI95[1:length(MultiNames)],
                    P_Value = P_Value[1:length(MultiNames)])

Multi.OS

#Recurrence-free survival
FML <- as.formula(paste0('Sur2~',paste(MultiNames,collapse = '+')))
MultiMod <- coxph(FML,data = cox.os)
SMultiMod <- summary(MultiMod)
HR <- round(SMultiMod$coefficients[,2],2)
P_Value <- round(SMultiMod$coefficients[,5],3)
LCI <- round(SMultiMod$[conf.int](https://nam12.safelinks.protection.outlook.com/?url=http%3A%2F%2Fconf.int%2F&data=05%7C01%7Ccaroline.johnson%40yale.edu%7C4c483d99d8444b6f17de08da610f3a81%7Cdd8cbebb21394df8b4114e3e87abeb5c%7C0%7C0%7C637929015283336878%7CUnknown%7CTWFpbGZsb3d8eyJWIjoiMC4wLjAwMDAiLCJQIjoiV2luMzIiLCJBTiI6Ik1haWwiLCJXVCI6Mn0%3D%7C3000%7C%7C%7C&sdata=FIiYgQkxa293r7YkFqNxzxTEqBGZKBuSBwzmKf1IJao%3D&reserved=0)[,3],2)
UCI <- round(SMultiMod$[conf.int](https://nam12.safelinks.protection.outlook.com/?url=http%3A%2F%2Fconf.int%2F&data=05%7C01%7Ccaroline.johnson%40yale.edu%7C4c483d99d8444b6f17de08da610f3a81%7Cdd8cbebb21394df8b4114e3e87abeb5c%7C0%7C0%7C637929015283336878%7CUnknown%7CTWFpbGZsb3d8eyJWIjoiMC4wLjAwMDAiLCJQIjoiV2luMzIiLCJBTiI6Ik1haWwiLCJXVCI6Mn0%3D%7C3000%7C%7C%7C&sdata=FIiYgQkxa293r7YkFqNxzxTEqBGZKBuSBwzmKf1IJao%3D&reserved=0)[,4],2)
CI95 <- paste(LCI,'-',UCI)
Multi.RFS <- data.frame(Characteristics = MultiNames[1:length(MultiNames)],
                    Hazard_Ratio = HR[1:length(MultiNames)],
                    CI95 = CI95[1:length(MultiNames)],
                    P_Value = P_Value[1:length(MultiNames)])

Multi.RFS
